# Identification of differentially expressed proteins in spontaneous thymic lymphomas from knockout mice with deletion of p53

**DOI:** 10.1186/1477-5956-6-18

**Published:** 2008-06-10

**Authors:** Bent Honoré, Søren Buus, Mogens H Claësson

**Affiliations:** 1 Institute of Medical Biochemistry, University of Aarhus, Ole Worms Allé, Bldg. 1170, DK-8000 Aarhus C, Denmark; 2 Laboratory of Experimental Immunology, Faculty of Health Sciences, University of Copenhagen, Denmark

## Abstract

**Background:**

Knockout mice with a deletion of p53 spontaneously develop thymic lymphomas. Two cell lines (SM5 and SM7), established from two independent tumours, exhibited about fifty to seventy two-fold differentially expressed proteins compared to wild type thymocytes by two-dimensional gel electrophoresis (2D-PAGE).

**Results:**

Protein spots excised from 2D-PAGE gels, were subjected to *in-gel *tryptic digestion and identified by liquid chromatography – tandem mass spectrometry. A total of 47 protein spots were identified. Immunological verification was performed for several of the differentially regulated proteins where suitable antibodies could be obtained. Functional annotation clustering revealed similarities as well as differences between the tumours. Twelve proteins that changed similarly in both tumours included up-regulation of rho GDP-dissociation inhibitor 2, proteasome subunit α type 3, transforming acidic coiled-coil containing protein 3, mitochondrial ornithine aminotransferase and epidermal fatty acid binding protein and down-regulation of adenylosuccinate synthetase, tubulin β-3 chain, a 25 kDa actin fragment, proteasome subunit β type 9, cofilin-1 and glia maturation factor γ.

**Conclusion:**

Some of the commonly differentially expressed proteins are also differentially expressed in other tumours and may be putative diagnostic and/or prognostic markers for lymphomas.

## Background

The p53 protein is a ubiquitous transcription factor that is normally expressed at very low levels. In a majority of spontaneous virally- or chemically-transformed tumours, however, p53 protein expression increased. Single nucleotide mutations may result in transcription/translation of p53 protein without transcriptional nuclear activity. Consequently, mutated p53 protein accumulates in the cytoplasm where it is proteolytically fragmented. p53-derived peptides are presented by the MHC class I molecules to the immune system thereby functioning as potential tumour rejection epitopes [1].

Functionally, p53 protein halts proliferating cells in the G_1 _phase, where DNA repair occurs. In addition, p53 prevents mutations in newly synthesized DNA [2-6]. Individuals genetically deficient in p53, due to mutations or gene deletion, are more susceptible to the development of spontaneous tumours [5-7]. These data are consistent with the high frequency of tumours, in particular thymic lymphomas, observed in p53-/- mice from the 12th week of age [8].

Previously, we have analyzed transcript [8,9] and protein profiles [10] of wild type mice thymocytes and compared these with corresponding profiles of two independently established thymic lymphoma cell lines, SM5 and SM7. These cell lines spontaneously arose in p53 deleted knockout mice. Based upon 2D-PAGE differential analysis, we identified significant changes in two protein spots (in p53 deleted cells) before tumour formation. The vast majority of changes, however, occurred after tumour formation. The SM5 cell line exhibited about seventy protein spot changes while SM7 displayed about fifty spot changes [10].

The aim of the present study was to establish the identity of these tumor-associated differentially expressed proteins. Using *in-gel *tryptic digestion and liquid chromatography – electrospray ionization – tandem mass spectrometry [11] we here report the identification of 47 of these protein spots.

## Results

### Differentially regulated proteins in thymic lymphomas

**Figure 1:**
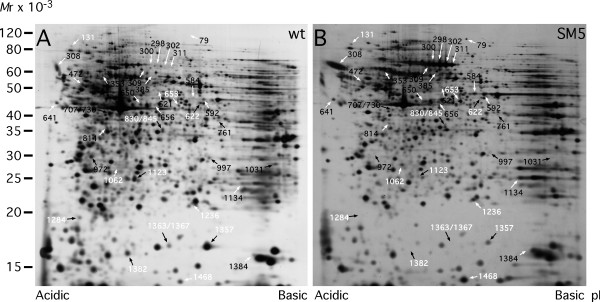
Two-dimensional gels of wt thymocytes (A) and of SM5 cells from a spontaneous derived tumour (B). Identified proteins that change more than two-fold between wt thymocytes and the SM5 tumour cells are shown based upon previously detected differentially expressed protein spots [10]. Up-regulated proteins are shown with white arrows and down-regulated proteins with black arrows. Proteins that change similarly in the SM5 and SM7 tumour cells are indicated with white numbers. The identified proteins are listed in Tables 1 and 2. For alignment purposes the Canvas program was used for mutual adjustment of gels.

**Figure 2:**
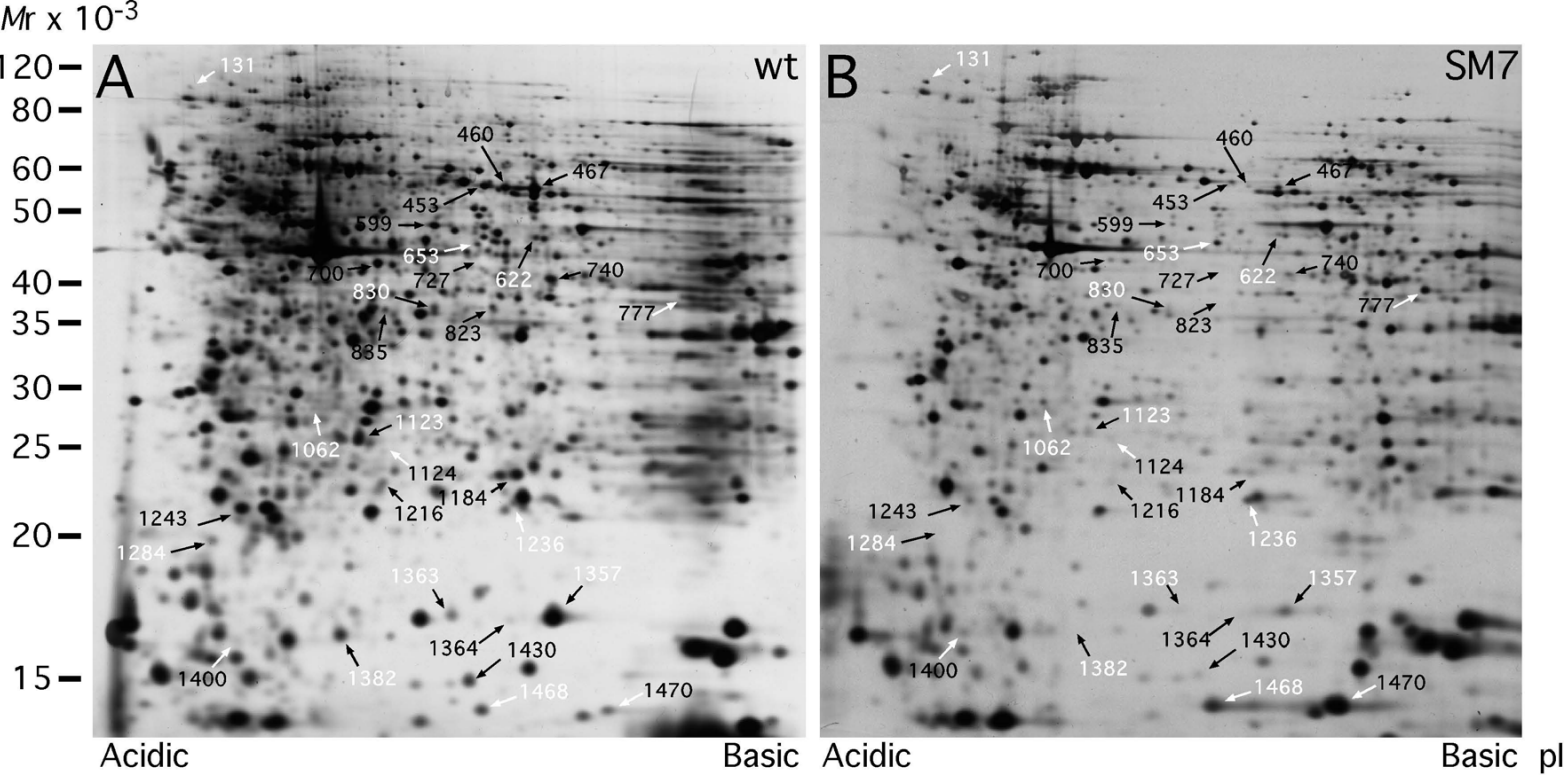
Two-dimensional gels of wt thymocytes (A) and of SM7 cells from a spontaneous derived tumour (B). Identified proteins that change more than two-fold between wt thymocytes and the SM7 tumour cells are shown based upon previously detected differentially expressed protein spots [10]. Up-regulated proteins are shown with white arrows and down-regulated proteins with black arrows. Proteins that change similarly in the SM5 and SM7 tumour cells are indicated with white numbers. The identified proteins are listed in Tables 1 and 3. For alignment purposes the Canvas program was used for mutual adjustment of gels.

Figs. 1 and 2 display the gel localization of the proteins identified in the present study. Twelve protein spots, differentially expressed in both tumour cell lines (i.e. SM5 and SM7) are labelled using white numbering. Protein spots that were uniquely differentially expressed in SM5 or SM7 cells are labelled using black numbering. Protein identifications are listed in Tables 1, 2 and 3. Of the 47 identified spots 5, i.e. 472, 521, 707/730, 1216 and 1470 were ambiguously determined, since more than one protein were present in each spot. Thus, for these 5 spots it could not be unequivocally determined which of the proteins that were differentially expressed. Western blotting was used to verify several of the changes seen.

**Table 1:** Differentially regulated proteins commonly found in two different tumours, SM5 and SM7

Protein	Identification	Peptide sequence	Charge	Mascot Score	Mr (Da)	pI	SM5 versus Wt^a^	SM7 versus Wt^b^
1236	Rho GDP-dissociation inhibitor 2/Ly-GDI GDIS_MOUSE	LNYKPPPQK (20–28)	2	116	21,754	6.81	Up	Up
		TLLGDVPVVADPTVPNVTVTR (49–69)	2					
		YVQHTYR (123–129)	2					
1062	Proteasome subunit α type 3 PSA3_MOUSE	SSIGTGYDLSASTFSPDGR (1–19) (Acetyl N-term)	2	112	27,861	5.37	Up	Up
		AVENSSTAIGIR (29–40)	2					
131	Transforming acidic coiled-coil containing protein 3/ARNT-interacting protein TACC3_MOUSE	GLLPAEPIVDVLK (416–428)	2	31*	88,566	4.04	Up	Up
		AQAEVLALQASLR (582–594)	2					
653	Ornithine aminotransferase, mitochondrial OAT_MOUSE	TEQGPPSSEYIFER (33–46)	2	147	43,842	6.56	Up	Up
		LFNYNK (130–135)	2					
		LPSDVVTSVR (363–372)	2					
		ESVEIINK (427–434)	2					
1468	Fatty acid binding protein, epidermal FABPE_MOUSE	FDETTADGR (72–80)	2	66	14,264	6.62	Up	Up
622	Adenylosuccinate synthetase PURA2_MOUSE	VVDLLAQDADIVCR (46–59)	2	140	45,185	6.95	Up	Up
		ELPVNAQNYVR (420–430)	2					
		FIEDELQIPVK (431–441)	2					
830/845	Tubulin β-3 chain TBBX_MOUSE^c^	AILVDLEPGTMDSVR (63–77)	2	36	36,980/36,445	6.26/6.25	Up	Up
1123	Actin (C-terminal) ACTX_MOUSE^d^	DLTDYLMK (184–191)	2	43	25,609	5.70	Down	Down
		EITALAPSTMK (316–326)	2					
1284	Proteasome subunit β type 9/LMP-2d PSB9_MOUSE	VSAGTAVVNR (40–49)	2	129	19,837	4.42	Down	Down
		FTTDAITLAMNR (174–185)	2					
		VILGDELPK (207–215)	2					
1357	Cofilin-1 COF1_MOUSE	YALYDATYETK (81–91)	2	65/65	17,159	7.16	Down	Down
1363/1367	Cofilin-1 COF1_MOUSE	YALYDATYETK (81–91)	2	20**	17,199	6.38	Down	Down
1382	Glia maturation factor γ GMFG_MOUSE	FVVYSYK (68–74)	2	117/75	16,626	5.56	Down	Down
		LVQTAELTK (111–119)	2					
		TTDDLTETWLK (125–135)	2					

The significance level for the peptides given were p < 0.05 when searching in the total database except the protein indicated with (*) that was significant when searching in the mouse part of the database and the peptide given with (**) that was not significant. The non-significant peptide (1363/1367) was included because of the similarity to the significant peptide 1357.

^a ^Up, protein that is up-regulated in SM5 cells versus wt. Down, protein that is down-regulated in SM5 cells versus wt.

^b ^Up, protein that is up-regulated in SM7 cells versus wt. Down, protein that is down-regulated in SM7 cells versus wt.

^c ^X is 2A, 2B, 3 or 5

^d ^X is A, B, C, G, H or S

**Table 2:** Differentially regulated proteins in the SM5 tumour

Protein	Identification	Sequence	Charge	Mascot Score	Mr	pI	SM5 versus wt^a^
79	Vinculin VINC_MOUSE	SLGEIAALTSK (433–443)	2	29*	101,649	6.64	Up
298/300/302/309/311	Albumin ALBU_BOVIN	LVNELTEFAK (66–75)	2	569/623/943/164/505	64.8–65.6 (kDa)	5.92–6.37	Up
		SLHTLFGDELCK (89–100)	2				
		LKPDPNTLCDEFKADEK (139–155)	3				
		YLYEIAR (161–167)	2				
		LSQKFPK (242–248)	2				
		AEFVEVTK (249–256)	2				
		YICDNQDTISSK (286–297)	2				
		SHCIAEVEK (310–318)	2				
		EYEATLEECCAK (375–386)	2				
		HLVDEPQNLIK (402–412)	2				
		LGEYGFQNALIVR (421–433)	2				
		KVPQVSTPTLVEVSR (437–451)	2/3				
		VPQVSTPTLVEVSR (438–451)	2				
		LCVLHEK (483–489)	2				
		CCTESLVNR (499–507)	2				
		QTALVELLK (549–557)	2				
		TVMENFVAFVDK (569–580)	2				
		CCAADDKEACFAVEGPK (581–597)	3				
308	α-2HS-glycoprotein FETUA_BOVIN	HTLNQIDSVK (58–67)	2	519	63.2 (kDa)	3.58	Up
		QQTQHAVEGDCDIHVLK (104–120)	3				
		QDGQFSVLFTK (121–131)	2				
		CDSSPDSAEDVR (132–143)	2				
		CDSSPDSAEDVRK (132–144)	2/3				
		EVVDPTKCNLLAEK (212–225)	3				
		CNLLAEK (219–225)	2				
		ALGGEDVR (238–245)	2				
		TPIVGQPSIPGGPVR (334–348)	2				
353	Serine/threonine-protein phosphatase 2A 65 kDa regulatory subunit A α isoform 2AAA_MOUSE	LAGGDWFTSR (134–143)	2	138/70	61,309	4.97	Up
		MAGDPVANVR (527–536)	2				
		LTQDQDVDVK (566–575)	2				
385	Prolyl 4-hydroxylase α-1 subunit P4HA1_MOUSE	LQDTYNLDTNTISK (137–150)	2	61/140	59,705	6.06	Up
		RPCTLSELE (526–534)	2				
521	Ig α-1 chain C region IGHA1_HUMAN/Lysozyme C LYSC_HUMAN/Deleted in malignant brain tumours 1 protein DMBT1_HUMAN	DASGVTFTWTPSSGK (154–168)	2	181	50,620	6.15	Up
		WLQGSQELPR (264–273)	2				
		QEPSQGTTTFAVTSILR (283–299)/	2				
		STDYGIFQINSR (69–80)/	2	87			
		FGQGSGPIVLDDVR (73–86)	2	77			
584	α-enolase ENOA_Mouse	GNPTVEVDLYTAK (15–27)	2	241	47,174	6.83	Up
		AAVPSGASTGIYEALELR (32–49)	2				
		YITPDQLADLYK (269–280)	2				
		VNQIGSVTESLQACK (343–357)	2				
		YNQILR (406–411)	2				
		IEEELGSK (412–419)	2				
592	α-enolase ENOA_Mouse	AAVPSGASTGIYEALELR (32–49)	2	179	46,971	7.03	Up
		YITPDQLADLYK (269–280)	2				
		DATNVGDEGGFAPNILENK (202–220)	2				
641	ERC-55 RCN2_MOUSE	VIDFDENTALDDTEEGSFR (133–151)	2	31*	44,221	3.51	Up
650	Eukaryotic translation initiation factor 3 subunit 4 IF34_MOUSE	VTNLSEDTR (243–251)	2	53	44,126	5.83	Up
814	Eukaryotic translation initiation factor 2 subunit 1 IF2A_MOUSE	VVTDTDETELAR (276–287)	2	81	37,463	5.12	Up
1134	Triosephosphate isomerase TPIS_MOUSE	VVLAYEPVWAIGTGK (159–173)^b^	2	37	25,173	8.28	Up
1384	Nucleoside diphosphate kinase B NDKB_MOUSE	GDFCIQVGR (91–99)	2	46	16,433	8.66	Up
472	UV excision repair protein RAD23 homolog B RD23B_MOUSE/Endoplasmin ENPL_MOUSE	IDIDPEETVK (15–24)	2	51	53,046	4.41	Up
		ASFNNPDR (213–220)/	2				
		SILFVPTSAPR (305–315)	2	83			
		GVVDSDDLPLNVSR (355–368)	2				
707/730	Actin ACTB_MOUSE/ UBA/UBX 33.3 kDa protein U33K_MOUSE	AGFAGDDAPR (1–10)	2	156	41,158/40,562	5.19/5.23	Down
		GYSFTTTAER (179–188)	2				
		SYELPDGQVITVGNER (218–233)/ LPDGTSLTQTFR (220–231)	2	74			
761	Poly(rC)-binding protein 1 PCBP1_MOUSE	INISEGNCPER (47–57)	2	110	39,587	7.46	Down
		IANPVEGSSGR (315–325)	2				
972	Proteasome inhibitor PI31 subunit PSMF1_MOUSE	VLIDPSSGLPNR (220–231)	2	43	31,039	4.68	Down
997	Histone H4 H4_MOUSE	ISGLIYEETR (46–55)	2	32*	30,490	7.22	Down
1031	ATP synthase α chain, mitochondrial ATPA_MOUSE	VLSIGDGIAR (74–83)	2	86	29,286	9.32	Down
		VVDALGNAIDGK (150–161)	2				

The significance level for the peptides given were p < 0.05 when searching in the total database except the peptides indicated with (*) that were significant when searching in the mouse part of the database.

^a ^Up, protein that is up-regulated in SM5 cells versus wt. Down, protein that is down-regulated in SM5 cells versus wt.

^b ^The peptide was not given as bold in the search, but was similar to a bold peptide in various other species except that L was exchanged with I.

**Table 3:** Differentially regulated proteins in the SM7 tumour

Protein	Identification	Sequence	Charge	Mascot score	Mr	pI	SM7 versus wt^a^
777	40 kDa peptidyl-prolyl cis-trans isomerase PPID_MOUSE	TLENVEVNGEKPAK (160–173)	2	66	39,459	5.23	Up
		ILLISEDLK (218–226)	2				
		EYDQALADLK (321–330)	3				
1124	Acyl-protein thioesterase 1 LYPA1-MOUSE	ASFQGPINSANR (150–162)	2	54	25,565	5.88	Up
1400	NudC domain- containing protein 2 NUDC2_MOUSE	AQDIQCGLQSR (40–50)	2	57	16,128	4.76	Up
1470	40S ribosomal protein S12 RS12_MOUSE/Galectin-7LEG7_MOUSE	AEEGIAAGGVMDVNTALQEVLK (1–22) (Acetyl N-term)	2	125	14,314	7.83	Up
		LGEWVGLCK (84–92)/	2				
		LTDSEVVFNTK (54–64)	2	81			
467	Coronin-1A COR1A_MOUSE	VSQTTWDSGFCAVNPK (30–45)	2	267	54,369	6.95	Down
		DGALICTSCR (187–196)	2				
		ILTTGFSR (234–241)	2				
		QVALWDTK (246–253)	2				
		DAGPLLISLK (384–393)	2				
		ATPEPSGTPSSDTVSR (417–432)	2				
453	Coronin-1A COR1A_MOUSE	ADQCYEDVR (21–29)	2	123	54,503	6.59	Down
		VSQTTWDSGFCAVNPK (30–45)	2				
		DAGPLLISLK (384–393)	2				
460	Coronin-1A COR1A_MOUSE	VSQTTWDSGFCAVNPK (30–45)	2	185	54,503	6.74	Down
		ILTTGFSR (234–241)	2				
		DAGPLLISLK (384–393)	2				
		ATPEPSGTPSSDTVSR (417–432)	2				
700	Adenosine deaminase ADA_MOUSE	AQTPAFNKPK (1–10) (Acetyl N-term)	2	130/188	41,426	5.83	Down
		GIALPADTVEELR (34–46)	2				
		IAYEFVEMK (81–89)	2				
		EGVVYVEVR (93–101)	2				
		ANYSLNTDDPLIFK (288–301)	2				
727	Heterogeneous nuclear ribonucleoprotein A/B ROAA_MOUSE	IFVGGLNPEATEEK (161–174)	2	15**	40,430	6.69	Down
823	4-hydroxyphenyl puruvate dioxygenase HPPD_MOUSE	TEDIITAIR (270–278)	2	28*	36,980	6.66	Down
835	Eukaryotic translation initiation factor 3 subunit 2 IF32_MOUSE	SYSSGGEDGYVR (299–310)	2	56/51	36,622	5.89	Down
1216	Ras-related protein Rab-11B RB11B_MOUSE/ATP-dependent RNA helicase DDX39 DDX39_MOUSE	GAVGALLVYDIAK (82–94)	2	65	22,463	5.87	Down
		NILTEIYR (166–173)/	2				
		ILVATNLFGR (338–348)	2	90			
1243	Peroxiredoxin-2 PRDX2_MOUSE	GLFIIDAK (128–135)	2	106	21,457	4.76	Down
		QITVNDLPVGR (140–150)	2				
1364	ADP-ribosylation factor 1 ARFX_MOUSE^b^	ILMVGLDAAGK (19–29)	2	50	17,059	6.80	Down
1430	Ubiquitin-conjugating enzyme E2 N UBE2N_MOUSE	LLAEPVPGIK (15–24)	2	107/128	15,088	6.53	Down
		TNEAQAIETAR (131–141)	2				
599	Actin-like protein 3 ARP3_MOUSE	YSYVCPDLVK (231–240)	2	97	46,669	6.25	Down
		NIVLSGGSTMFR (318–329)	2				
		LSEELSGGR (349–357)	2				
		DYEEIGPSICR (399–409)	2				
740	Heterogeneous nuclear ribonucleoprotein A/B ROAA_MOUSE	IFVGGLNPEATEEK (161–174)	2	113	40,104	7.11	Down
		EVYQQQQYGSGGR (238–250)	2				
1184	Transforming protein RhoA RHOA_MOUSE	LVIVGDGACGK (8–18)	2	79	23,410	6.82	Down
		IGAFGYMECSAK (151–162)	2				

The significance level for the peptides given were p < 0.05 when searching in the total database except the peptide indicated with (*) that was significant when searching in the mouse part of the database and the peptide given with (**) that was not significant. The non-significant peptide (727) was included because of the similarity to the significant peptide 740.

^a ^Up, protein that is up-regulated in SM7 cells versus wt. Down, protein that is down-regulated in SM7 cells versus wt.

^b ^X is 1, 2, 3, 4 or 5.

### Commonly differentially regulated proteins

The commonly regulated proteins are given in Table 1. We identified up-regulation of rho GDP-dissociation inhibitor 2 (1236), proteasome subunit α type 3 (1062), transforming acidic coiled-coil containing protein 3 (131), mitochondrial ornithine aminotransferase (653) and epidermal fatty acid binding protein (1468).

Among the down regulated proteins, we identified adenylosuccinate synthetase (622), tubulin β-3 chain (830/845), a 25 kDa actin fragment (1123), proteasome subunit β type 9 (1284), basic cofilin-1 variant (1357), acidic cofilin-1 variant (1363/1367) and glia maturation factor γ, (1382).

### Uniquely differentially regulated proteins

Tables 2 and 3 show the obtained peptides from uniquely regulated proteins. Unambiguously identified up-regulated proteins in tumour cells SM5 included: vinculin, serine/threonine-protein phosphatase 2A 65 kDa regulatory subunit A α isoform, prolyl 4-hydroxylase α-1 subunit, α enolase, ERC-55, eukaryotic translation initiation factor 3 subunit 4, eukaryotic translation initiation factor 2 subunit 1, triosephosphate isomerase, nucleoside diphosphate kinase B and in tumour cells SM7: 40 kDa peptidyl-prolyl cis-trans isomerase, acyl-protein thioesterase 1, and nudC domain-containing protein 2. Spots 298, 300, 302, 309 and 311 contained bovine albumin while spot 308 contained bovine α 2HS-glycoportein or fetuin. Spots 521, 472 and 1470 contained more than one protein.

Unambiguously identified down-regulated proteins in tumour cells SM5 included: poly(rC)-binding protein 1, proteasome inhibitor PI31 subunit, histone H4, ATP synthase α chain and in the SM7 tumour cell line: coronin-1A, adenosine deaminase, heterogeneous nuclear ribonucleoprotein A/B, 4-hydroxyphenyl puruvate dioxygenase, eukaryotic translation initiation factor 3 subunit 2, peroxiredoxin-2, ADP-ribosylation factor 1, ubiquitin-conjugating enzyme E2 N, actin-like protein 3 and transforming protein RhoA. Spots 707/730 and 1216 contained more than one protein.

### Immunological verification of differential regulation

**Figure 3:**
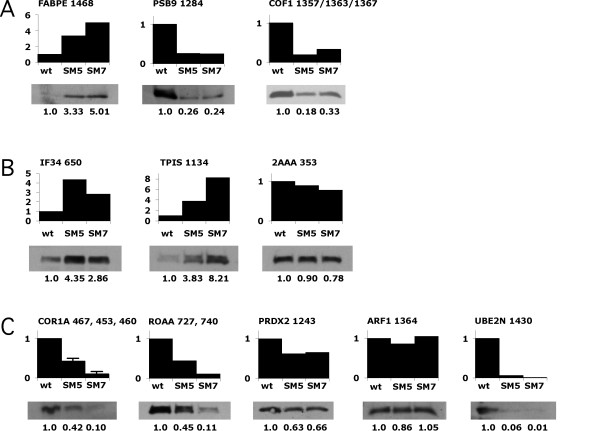
Western blots of wt thymocytes and thymocytes from two spontaneously arising tumours, SM5 and SM7. Equal amounts of protein were added to the lanes used for analysis of one antibody, either 5, 10 or 30 mg per lane. The bands were scanned and quantitated using the Quantity One program. The measured changes are indicated below the blot with wild type arbitrarily set to 1.0. Proteins that, from 2D-PAGE analysis, were found to change at least 2-fold are given in (A) for the commonly regulated and in (B) and (C) for the individually changed in SM5 and SM7, respectively.

To independently confirm 2D-PAGE data, 1D Western Blot analysis was used to assess the differential expression of selected protein immunoreactive. Ideally, a single strong band on a Western blot reflects an antibody possessing high affinity with specific reaction against the antigen. The data obtained by Western Blot analysis is presented in Fig. 3.

Fig. 3A depicts Western blot analysis for three commonly regulated antigens: i.e. epidermal fatty acid binding protein (FABPE), proteasome subunit β type 9 (PSB9) and cofilin-1 (COF1). Each produced strong bands in the cells where the proteins were present in high amounts. Furthermore the data are consistent with and confirm 2D-PAGE data. That is, that FABPE is more than 2-fold up-regulated in both of the tumour cells and that PSB9 and COF1 are both more than 2-fold down-regulated in tumour cells.

Fig. 3B and 3C depict Western blot data that verifies the differential regulation of selected uniquely regulated proteins. That is, eukaryotic translation initiation factor 3 subunit 4 (IF34) and triosephosphate isomerase (TPIS) were found to be more than 2-fold up-regulated in the SM5 cell line but in fact also in the SM7 cell line. Similarly, down-regulation in tumour cells SM7 was confirmed in case of the proteins coronin-1A (COR1A, 10-fold), heterogeneous nuclear ribonucleoprotein A/B (ROAA, 9-fold), peroxiredoxin-2 (PRDX2, 1.5-fold), ubiquitin-conjugating enzyme E2 N (UBE2N, >10-fold) and actin-like protein 3 (ARP3, 3.1-fold). All of these proteins were also down-regulated in the tumour SM5.

Based on Western blots data, 2 of the 11 proteins did not display concordance with 2D-PAGE data, i.e. serine/threonine-protein phsophatase 2A 65 kDa regulatory subunit A α isoform (2AAA) and ADP-ribosylation factor (ARF). In case of ADP-ribosylation factor, the apparent discrepancy between Western and 2D-PAGE data may be due to the fact that six isoforms are known of this protein [12]. It is highly unlikely that the six isoforms migrate in the same spot on the 2D gel. Generally, the 2D gel will be able to distinguish the isoforms as seen with cofilin-1 (1357 and 1363) and coronin-1A (453, 460, and 467) although the peptides obtained by MS do not contain sufficient information to determine which of the variants 1 to 5 is identified. Only variant 6 can be ruled out. The 1D Western blot, however, generally recognize all the isoforms in one band as seen with cofilin-1 and coronin-1A. The changed expression level of one isoform determined by 2D-PAGE may then be obscured if other isoforms do not change similarly or change in the opposite direction giving no major overall change of the different isoforms taken as a whole on the Western blot. The same may be true for serine/threonine-protein phsophatase 2A 65 kDa regulatory subunit A α isoform (2AAA) [13]. Thus, generally our study shows, as others [14,15], that the differential regulation of the proteins seen by 2D-PAGE could be quantitatively confirmed by Western blotting in 8 of 11 cases. These eight were epidermal fatty acid binding protein (FABPE), proteasome subunit β type 9 (PSB9), cofilin-1 (COF1), eukaryotic translation initiation factor 3 subunit 4 (IF34), triosephsophate isomerase (TPIS), coronin-1A (COR1A), heterogeneous nuclear ribonucleoprotein A/B (ROAA) and ubiquitin-conjugating enzyme E2 N (UBE2N).

With respect to the five spots containing more than one protein we tried antibodies against UV excision repair protein RAD23 homolog B (472), galectin-7 (1470) and Ras related protein Rab-11A (spot 1216). Unfortunately, the quality of the results was insufficient for firm conclusions.

## Discussion

Previously, we have detected differentially-expressed proteins in spontaneously formed tumours in p53 knockout mice [10]. Changes in the expression of two of the proteins identified preceded tumour formation. In the vast majority, however, changes in expression coincided with or paralleled tumour formation, with about seventy protein spots changing in the SM5 cell line while about fifty spot changes were found in the SM7 cell line. The aim of this study was to further characterise and identify, tumour-associated changes in protein expression.

A subset of the proteins were commonly regulated, changing at least two-fold by 2D-PAGE in both tumours while a number of proteins, originally detected in one of the tumours also could be found to be differentially regulated in the other tumour as detected by immunodetection. Such differentially expressed proteins may represent proteins that are of general importance in order to understand the transformation process initiated by the deletion of p53. Furthermore, some of them might be suitable as diagnostic or prognostic markers for lymphomas or other tumours that include deletion of p53. Several genes responsive to elevated levels of p53 have been identified [16-18]. In the p53 deleted cells we did not detect differential regulation of direct downstream targets of p53 within the major pathways including cell cycle arrest, DNA repair, apoptosis, angiogenesis or cellular stress response. We did observe down regulation of a C-terminal actin fragment in both cell lines (spot 1123). α actin has been reported to be a transcriptional target of p53 [19] and down-regulation could be due to a decreased expression of the actin gene. The observation might also be explained by a decrease in the cleavage of the actin protein.

### Functional annotation clustering of differentially regulated proteins

Each of the identified proteins may be functionally classified based on the Gene Ontology annotation system [20]. This system is a structured, organized vocabulary that systematically describes each protein with respect to cellular component, biological process and molecular function. By using the DAVID Bioinformatics Resource [21] we performed a functional annotation clustering of the proteins involved in the transformation. With the highest classification stringency, we found seven clusters for SM5 and eleven clusters for SM7 as shown in Tables 4 and 5. The clusters are listed with the most enriched at top. For example, the 3 proteins PURA2, NKDB and ATPA of the 24 proteins analyzed with the cell line SM5 belong to the cluster purine nucleotide biosynthetic process. The p value at 0.0039 indicates that this function is significantly over represented among the 24 proteins analyzed. Similarly, proteins involved in cellular process, glucose metabolic process, translation factor activity (nucleic acid binding) and the actin cytoskeleton were all significant. Some of the proteins belong to more than one category since they possess multiple functions. The SM7 cell line was in some respects similar to SM5 but in other respects different since functions that were over represented by the transformation included GTP binding, cytoskeleton, cellular process, ubiquitin-dependent protein catabolic process, intracellular transport and GTPase activity. The partly different functions of the two cells may partly explain the different physiology of the two cell lines where SM7 is 3–5 times more tumourigenic compared with SM5 [10].

**Table 4:** Functional annotation clustering of proteins involved in the transformation of tumour cell line SM5

Functional annotation clustering^a^	Function	Proteins involved	P value
Purine nucleotide biosynthetic process	The chemical reactions and pathways resulting in the formation of a purine nucleotide, a compound consisting of nucleoside (a purine base linked to a deoxyribose or ribose sugar) esterified with a phosphate moiety at either the 3' or 5'-hydroxyl group of its glycose moiety.	PURA2	0.0039
		NKDB	
		ATPA	
Cellular process	Processes that are carried out at the cellular level, but are not necessarily restricted to a single cell. For example, cell communication occurs among more than one cell, but occurs at the cellular level.	H4	0.047
		PSB9	
		TBB3	
		VINC	
		IF34	
		PURA2	
		ENOA	
		IF2A	
		ATPA	
		P4HA1	
		FABPE	
		COF1	
		TPIS	
		TACC3	
		PSA3	
		NDKB	
		ACTB	
		PCBP1	
		OAT	
Glucose metabolic process	The chemical reactions and pathways involving glucose, the aldohexose gluco-hexose. D-glucose is dextrorotatory and is sometimes known as dextrose; it is an important source of energy for living organisms and is found free as well as combined in homo- and hetero-oligosaccharides and polysaccharides.	TPIS	0.006
		ENOA	
		FABPE	
Translation factor activity, nucleic acid binding	Functions during translation by binding nucleic acids during polypeptide synthesis at the ribosome.	IF34	0.011
		IF2A	
		PCBP1	
Actin cytoskeleton	The part of the cytoskeleton (the internal framework of a cell) composed of actin and associated proteins. Includes actin cytoskeleton-associated complexes.	COF1	0.022
		VINC	
		ACTB	
ATP binding	Interacting selectively with ATP, adenosine 5'-triphosphate, a universally important coenzyme and enzyme regulator.	NDKB	0.48
		ATPA	
		ACTB	
Regulation of cellular process	Any process that modulates the frequency, rate or extent of cellular processes, those that are carried out at the cellular level, but are not necessarily restricted to a single cell. For example, cell communication occurs among more than one cell, but occurs at the cellular level.	COF1	0.76
		VINC	
		TACC3	
		IF2A	

^a ^For each cluster is listed the GO term possessing the highest enrichment score using the DAVID bioinformatics Resource [21]. In some cases this GO term was obsolete in which case the nearest well defined term was listed.

**Table 5:** Functional annotation clustering of proteins involved in the transformation of tumour cell line SM7

Functional annotation clustering^a^	Function	Proteins involved	P value
GTP binding	Interacting selectively with GTP, guanosine triphosphate.	TBB3	0.0073
		ARF1	
		PURA2	
		RHOA	
Cytoskeleton	Any of the various filamentous elements that form the internal framework of cells, and typically remain after treatment of the cells with mild detergent to remove membrane constituents and soluble components of the cytoplasm. The term embraces intermediate filaments, microfilaments, microtubules, the microtrabecular lattice, and other structures characterized by a polymeric filamentous nature and long-range order within the cell. The various elements of the cytoskeleton not only serve in the maintenance of cellular shape but also have roles in other cellular functions, including cellular movement, cell division, endocytosis, and movement of organelles	COF1	0.0012
		TBB3	
		TACC3	
		ARP3	
		RHOA	
		ACTB	
Cellular process	Processes that are carried out at the cellular level, but are not necessarily restricted to a single cell. For example, cell communication occurs among more than one cell, but occurs at the cellular level.	PSB9	0.047
		TBB3	
		PURA2	
		ROAA	
		PRDX2	
		ADA	
		LYPA1	
		FABPE	
		IF32	
		COF1	
		ARF1	
		TACC3	
		HPPD	
		RHOA	
		PSA3	
		PPID	
		ACTB	
		UBE2N	
		OAT	
Ubiquitin-dependent protein catabolic process	The chemical reactions and pathways resulting in the breakdown of a protein or peptide by hydrolysis of its peptide bonds, initiated by the covalent attachment of a ubiquitin moiety, or multiple ubiquitin moieties, to the protein.	PSB9	0.010
		PSA3	
		UBE2N	
Intracellular transport	The directed movement of substances within a cell.	TBB3	0.042
		ARF1	
		TACC3	
		RHOA	
GTPase activity	Catalysis of the reaction: GTP + H_2_O → GDP + phosphate.	TBB3	0.010
		ARF1	
		ROHA	
Cellular protein metabolic process	The chemical reactions and pathways involving a specific protein, rather than of proteins in general, occurring at the level of an individual cell. Includes protein modification.	PSB9	0.098
		COF1	
		TBB3	
		PSA3	
		PPID	
		IF32	
		UBE2N	
Intracellular protein transport	The directed movement of proteins in a cell, including the movement of proteins between specific compartments or structures within a cell, such as organelles of a eukaryotic cell.	ARF1	0.094
		TACC3	
		RHOA	
Negative regulation of cellular process	Any process that stops, prevents or reduces the frequency, rate or extent of cellular processes, those that are carried out at the cellular level, but are not necessarily restricted to a single cell. For example, cell communication occurs among more than one cell, but occurs at the cellular level.	TACC3	0.19
		RHOA	
		PRDX2	
Regulation of cellular process	Any process that modulates the frequency, rate or extent of cellular processes, those that are carried out at the cellular level, but are not necessarily restricted to a single cell. For example, cell communication occurs among more than one cell, but occurs at the cellular level.	COF1	0.53
		TACC3	
		RHOA	
		ROAA	
		PRDX2	
Ion binding	Interacting selectively with ions, charged atoms or groups of atoms.	PURA2	0.93
		HPPD	
		ADA	

^a ^For each cluster is listed the GO term possessing the highest enrichment score using the DAVID bioinformatics Resource [21]. In some cases this GO term was obsolete in which case the nearest well defined term was listed.

### Differentially regulated cancer related proteins

Oncogenesis is not a term within the Gene Ontology classification. However, some of the commonly regulated proteins are known from other studies also to be involved in carcinogenesis and will be briefly described.

#### Rho GDP-dissociation inhibitor 2 (1236)

Rho GDP-dissociation inhibitor 2 is preferentially expressed in lymphocytes [22] and found to be up-regulated in the two tumour cells analyzed. Recently, the protein was also found to be up-regulated in oral squamous cell carcinoma whereas the mRNA level was only very slightly up-regulated [23]. The Rho GDP-dissociation inhibitors play a role in the modulation of the activity of the Rho proteins. Overexpression leads to rounding up of cells and loss of stress fibers and focal contact sides [24].

#### Transforming acidic coiled-coil containing protein 3 (TACC3) (131)

TACC3 was found to be up-regulated in both tumour cell lines. The transcript has been previously found to be strongly present in testis, at low level in human thymus and very low levels in a number of tissues of the immune system while several cancer cell lines express up-regulated levels of the transcript [25]. Recently, Jung et al. have found TACC3 protein expression to be a prognostic marker of non-small cell lung carcinoma where 14.8% of the tumours expressed high levels [26]. The authors also found TACC3 expression to be correlated with p53 expression and that patients with high expression of both proteins had significantly poorer prognosis than patients with low-level expression of both [26].

#### Ornithine aminotransferase (653)

Ornithine aminotransferase (OAT) was found to be up-regulated in both tumour cell lines. Previously, it has been found by subtractive hybridisation that the transcript of OAT is up-regulated in hepatocellular carcinomas [27].

#### Epidermal fatty acid binding protein (1468)

E-FABP is a fatty acid binding protein that was originally found to be up-regulated in psoriatic skin [28]. Recently, it was found that E-FABP is up-regulated in head and neck squamous cell carcinomas and a number of carcinoma cell lines [29]. In addition, it was found that EpCAM, a tumour associated antigen, up-regulates E-FABP and furthermore that EpCAM is positively correlated with the grade of dysplasia being a negative prognostic factor for breast cancer patients [29].

#### Proteasome subunit β type 9 (LMP2)

The proteasome is a complex structure localized in the cytosol and composed of several different subunits. Some of the subunits are constitutively expressed while others, including LMP2, belong to the facultative subunits that may exchange with the constitutively expressed subunits as a result of immune stress [30]. Proteasome subunit β type 9 was down-regulated in the tumour cells analyzed here. It has previously been found that LMP2 generally is down-regulated in several types of tumours such as laryngeal squamous cell carcinoma [31], head and neck squamous cell carcinoma [32], renal cell carcinoma [33], as well as in a number of tumour cell lines including small-cell lung carcinoma, hepatocellular carcinoma, colon adenocarcinoma and basophilic leukemia [34].

### Plasma derived proteins

In SM5 cells we identified two proteins that were markedly present and clearly originated from bovine plasma, fetuin (308) and albumin (298, 300, 302, 309 and 311). Both proteins were detected from SM5 cells but were only vaguely seen in SM7 cells. The likely differences between the isoforms of BSA are various types of modifications that may occur, e.g. following oxidation [35]. These modifications include glycosylations at a number of Lys residues. Since the cells were treated similarly, it is unlikely that the presence of them in one of the cell lines is due to contamination obtained during the preparation of the cells. Endothelial cells have previously been shown to take up fetuin [36]. Recently, it was shown that fibroblasts are also able to perform this internalization and that the internalization is down-regulated by hypoxia [37]. The present study indicates that especially transformed thymocytes (SM5) may have gained the ability to perform this internalisation. We, however, cannot exclude that the proteins also could adhere to the surface of the cells rather than being internalized.

### Comparison with previous observed changes in mRNA in SM7

Using one of the early mouse oncogene arrays, Atlas mouse array I (Clontech) encompassing 588 oncogenes, we have previously identified several differentially regulated transcripts in the two tumour cell lines [8,9]. Only a few overlaps were found between the identified set of proteins differentially expressed here and the previously identified differentially regulated transcripts. These included nucleoside diphosphate kinase B, UV excision repair protein RAD23 homolog B, and ubiquitin regulating enzyme E2.

## Conclusion

We detected and identified several proteins, that were differentially regulated as a result of transformation following deletion of the p53 gene. Immunological verification of differential regulation was performed in 8 of 11 cases. Among the two cell types SM5 and SM7 that differed in their carcinogenic potential, we also found differences in the functional annotation clustering. A major part of the commonly regulated set of proteins have previously been found to be related to several other types of cancers indicating that they may play a role in the transformation process. Studies will be performed to investigate their putative role as diagnostic or prognostic biomarkers in human lymphomas. Biopsies could be histochemically analyzed using antibodies against the differentially expressed proteins in order to diagnostically and/or prognostically classify lymphomas. Putative up-regulated proteins might further be analyzed for their presence in the blood to be used as diagnostic and/or prognostic biomarkers.

## Methods

### Analysis of two-dimensional gels

The performance and analysis of two-dimensional gels have been described in detail previously [10]. Briefly, silver stained gels were scanned in the transmissive mode on a GS-710 Imaging Densitometer (Bio-Rad) using Quantity One. The 16-bit gray scale TIFF files were imported into Melanie II. Initially, protein spots were automatically defined and quantified. Spot intensities were expressed as relative volumes in percentages (%VOL) by integrating the optical density of each pixel in the spot area (VOL) and dividing with the sum of volumes of all spots detected. About 10–12 of the spots were used as landmarks. One of the gels was selected as reference gel and used to align and match the other gels. The quality of the match was critically evaluated and necessary editions and corrections done manually. Comparisons were performed between two groups of gels by selecting spots that differed more than two-fold between the groups. These data were exported to Excel for further statistical analysis using Student's *t*-test as previously described [10].

### Identification of proteins by liquid chromatography-tandem mass spectrometry (LC-MS/MS)

Protein identification by LC-MS/MS was performed as previsously described [38]. Briefly, previously run gels [10] containing protein spots selected for identification were re-hydrated in water. The cellophane sheets were peeled off so the protein spots could be excised from the gels. Proteins were *in-gel *digested with trypsin. Gel pieces were first dehydrated in acetonitrile, then dried and proteins reduced for 1 h at 56°C in 10 mM dithiotreitol (DTT) and 100 mM NH_4_HCO_3_. The solution was exchanged with 55 mM iodoacetamide in 100 mM NH_4_HCO_3 _for 45 min. Then it was washed in 100 mM NH_4_HCO_3_, dehydrated in acetonitrile, rehydrated in 100 mM NH_4_HCO_3_, dehydrated in acetonitrile, dried and swelled in digestion buffer (50 mM NH_4_HCO_3_, 5 mM CaCl_2 _and 12.5 ng/μl trypsin Gold (mass spectrometry grade; Promega, Madison, WI, USA). Digestion was performed overnight at 37°C and the peptides were extracted by 1 change of 20 mM NH_4_HCO_3 _and 3 changes of 5% formic acid in 50% acetonitrile. The sample was finally dried and the peptides resuspended in 12 μl of buffer A (water/acetonitrile/formic acid, 97.7/2/0.3, V/V/V). The peptides were separated on an inert nano LC system composed of a FAMOS micro autosampler, a Switchos micro column switching module and an Ultimate micro pump from LC Packings (San Francisco, CA) before MS analysis. Of the *in-gel *digested samples 5 μl was preconcentrated and desalted on a 300 μm inner diameter × 5 mm Nano-Precolumn (LC Packings) packed with 5 μm C18 PepMap100 material. A 75 μm inner diameter × 15 cm Nano column packed with 3 μm C18 PepMap100 material was used to separate the peptides. Elution from the column was made with a gradient by mixing decreasing volumes of buffer A with increasing volumes of buffer B (water/acetonitrile/formic acid, 9.7/90/0.3, V/V/V). The peptides were eluted into the nano electrospray ion source of the quadrupole time-of-flight Q-TOF Ultima mass spectrometer (Micromass, Manchester, UK). MS survey scans were acquired using MassLynx 4 SP4 (Waters) at a rate of 1 per second from m/z 400–2000. The instrument was operated in a data-dependent MS to MS/MS switching mode. Doubly, triply and quadruply charged peptide ions detected in MS survey scans triggered a switch to MS/MS for obtaining peptide fragmentation spectra with an interval of *m/z *values 50–2000. Raw data were processed using ProteinLynx GlobalServer 2.1 (Waters). Processing parameters were as follows: Background Subtract: Normal, Background Threshold: 35%, Background Polynomial: 5, Smoothing Type: Savitzky-Golay, Smoothing Iterations: 2, Smoothing Window: 2 channels, Deisotoping Type: Normal, Deisotoping Threshold: 1%. The processed data were used to search the total part and the mouse fraction of the Swiss-Prot database (releases 49.7, 54.0 and 54.4) using the on-line version of the Mascot MS/MS Ion Search facility (Matrix Science, Ltd., ) [39]. Searching was performed with doubly and triply charged ions with 2 missed cleavages, a peptide tolerance of 20, 50 or 100 ppm, one variable modification either Carbamidomethyl-C or N-terminal acetyl and an MS/MS tolerance of 0.05 or 0.08 Da. Contaminating peptides, including keratins and trypsin were disregarded. At least one 'bold red' peptide was required in the search. Peptides for proteins with scores giving a less than 5% probability that the observed match was a random event are reported.

### Western blotting

Normal C57BL/6 mice (wt) were purchased from Bomholtgaard, Ry, Denmark. The mice were kept under conventional conditions at the animal facility at the Panum Institute. The thymus was pressed through a metal net and single cell suspensions were recovered on a foetal calf serum gradient allowing clumps and debris to settle. By haemocytometer examination more than 99% of the cells are lymphoid. Thymocytes were prepared from three mice and pooled. They were then washed extensively in PBS and lyophillized. C57BL/6J-Trp53tm1Tyj mice deficient for the p53 gene were purchased from the Jackson Laboratory, USA. Two spontaneously developing thymic lymphomas, SM5 and SM7, were explanted, in vitro cultured, and established as cell lines growing in RPMI-1640 culture medium supplemented with 10% fetal calf serum and 50 μM 2-mercaptoethanol. Cells were washed extensively in PBS, harvested with a rubber police man and lyophilized. Protein concentration was determined using a non-interfering assay (NI Protein Assay, Geno Technology Inc., St Louis, MO, USA). For 1D gel electrophoresis equal amounts of proteins (either 5, 10 or 30 μg) from each of the cell preparations, wt, SM5 and SM7 were added to each lane of a 10–20% Tris-Glycine gel (Invitrogen). After electrophoresis the separated proteins were transferred to a nitrocellulose membrane. For immunodetection, the membrane was incubated overnight at 4°C in PBS containing 0.05% Tween-20 and 5% skim milk, washed three times in PBS with Tween-20, and incubated with the appropriate dilution of the primary antibody for at least 1 h at room temperature in PBS with Tween-20 and 5% skim milk. After three washes in PBS with Tween-20 the blot was incubated for 1 h with peroxidase-conjugated secondary antibody in PBS with Tween-20. Finally, after five washes in PBS with Tween-20, the blot was developed using enhanced chemiluminescence (Amersham Biosciences, Piscataway, NJ, USA). The bands were visualized on film (Kodak), scanned with a GS-710 Imaging Densitometer from Bio-Rad and the intensities of the bands were evaluated using the Quantity One software package.

Several antibodies were obtained from commercial suppliers. The following rabbit antibodies were used: anti-cofilin (Abcam), dilution 1:10,000 (9 μg/ml); anti-ubiquitin conjugating enzyme E2 N (Calbiochem), dilution 1:10 (50 μg/ml); anti-proteasome 20S LMP2 (Abcam), dilution 1:100 (1 μg/ml); anti-peroxiredoxin 2 (Abcam), dilution 1:2,000 (0.5 μg/ml); anti-transforming acidic coiled coil containing protein 3 (Biolegend), dilution 1:5; anti-NM23-H1/H2 (Alexis) dilution 1:100 (10 μg/ml); anti-RAD23B (Abgent), dilution 1:10 (25 μg/ml), anti-cyclophillin 40 PPID (Abcam), dilution 1:10; anti-galectin 7 (Abcam), dilution 1:30 (33 μg/ml); anti-adenosine deaminase (ADA) (Nordic Biosite), dilution 1:20; anti-Rab11a (Nordic Biosite), dilution 1:60 (2 μg/ml); anti-Ly-GDI (Santa Cruz Biotechnology, INC.), dilution 1:50 (4 μg/ml); anti-serine/threonine protein phosphatase 2a/a (Sigma), dilution 1:10 (10 μg/ml). The following mouse antibodies were used: anti-ADP ribosylation factor (Abcam), dilution 1:50; anti-proteasome 20S α 3 (Abcam), dilution 1:25; anti-Actin like protein 3 (Arp3) (BD Biosciences Pharmingen), dilution 1:100 (2.5 μg/ml); anti-hnRNP-A2/B1 (ImmuQuest, Ltd.) dilution 1:500 (2 μg/ml); anti-vinculin (Abcam), dilution 1:10 (1 mg/ml); anti-Rho GDP-dissociation inhibitor 2 (Rho-GDI2) (Alexis) dilution 1:10 (50 μg/ml). The following goat antibodies were used: anti-coronin 1 (Everest Biotech), dilution 1:100 (5 μg/ml); anti-FABP5 (R & D Systems), dilution 1:10 (20 μg/ml); anti-triosephosphate isomerase (TPI1) (Everest Biotech, Ltd.), dilution 1:1,000 (0.5 μg/ml); anti-eIF3β (Santa Cruz Biotechnology, Inc.), dilution 1:5 (40 μg/ml). The following chicken antibody was used: anti-eukaryotic translation initiation factor 3, subunit 4 δ, 44 kDa (EIF3S4), (GenWay Biotech, Inc.), dilution 1:1,000 (1 μg/ml). Secondary HRP conjugated antibodies were purchased from DAKO (Denmark): anti-rabbit PO217, dilution 1:5,000 (0.26 μg/ml); anti-mouse PO260, dilution 1:5,000 (0.26 μg/ml); anti-goat, PO449, dilution 1:5,000 (0.1 μg/ml) and from Abcam (UK) anti-chicken (ab16349), dilution 1:2,500. Imaging Kodak films were scanned in the transmissive mode on a GS-710 Calibrated Imaging Densitometer from Bio-Rad using the Quantity One software package. Each band was thereby designated a value proportional to the protein concentration in the band. The quality of the antibody reactions was evaluated on the basis of the Western blots where a single strong band indicates a high-affinity specific antibody. Several antibodies were found to give Western blots of insufficient quality in our system either because reactions were to weak or because of the presence of several bands indicating that the antibody possesses low-affinity and/or unspecific reactivity in the given system.

## Competing interests

The authors declare that they have no competing interests.

## Authors' contributions

BH conceived the study, carried out the mass spectrometry determinations and immunoblotting analyses, interpreted the data and drafted the manuscript, SB participated in interpretation of data and revised the manuscript critically, MHC conceived the study, performed the cell preparations and revised the manuscript critically. All authors read and approved the final manuscript.
